# Bone apatite anisotropic structure control *via* designing fibrous scaffolds

**DOI:** 10.1039/d0ra01295e

**Published:** 2020-04-02

**Authors:** Sungho Lee, Fukue Nagata, Katsuya Kato, Takayoshi Nakano

**Affiliations:** National Institute of Advanced Industrial Science and Technology 2266-98 Anagahora, Shimoshidami, Moriyama-ku Nagoya 463-8560 Japan sungho.lee@aist.go.jp; Division of Materials and Manufacturing Science, Graduate School of Engineering, Osaka University 2-1 Yamadaoka, Suita Osaka 565-0871 Japan nakano@mat.eng.osaka-u.ac.jp

## Abstract

Bone tissue has an anisotropic structure, associated with the collagen fibrils' orientation and the *c*-axis direction of the bone apatite crystal. The bone regeneration process comprises two main phases: bone mineral density restoration (bone quantity), and subsequent recovery of bone apatite *c*-axis orientation (bone quality). Bone quality is the determinant factor for mechanical properties of bone. Control of osteoblast alignment is one of the strategies for reconstructing bone quality since the collagen/apatite matrix orientation in calcified tissues is dependent on the osteoblast orientation. In this work, fibrous scaffolds designed for reconstruction of bone quality *via* cell alignment control was investigated. The fibrous scaffolds were fabricated using the electrospinning method with poly(lactic acid) at various fiber collecting speeds. The degree of fiber alignment in the prepared fibrous scaffolds increased with increasing fiber collecting speed, indicating that the fibers were oriented in a single direction. The alignment of osteoblasts on the fibrous scaffolds as well as the subsequent apatite *c*-axis orientation increased with increasing fiber collecting speed. We successfully controlled cell alignment and apatite *c*-axis orientation using the designed morphology of fibrous scaffolds. To the best of our knowledge, this is the first report demonstrating that adjusting the degree of fiber orientation for fibrous scaffolds can manipulate the regeneration of bone quality.

## Abstract 2

Osteoblast alignment on the fibrous scaffolds as well as the subsequent apatite *c*-axis orientation increased with increasing fiber collecting speed. We successfully controlled cell alignment and apatite *c*-axis orientation using the designed morphology of fibrous scaffolds.

## Graphical abstract

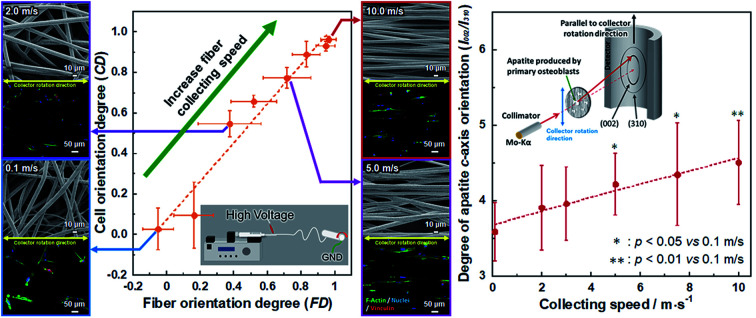

## 1. Introduction

Bone has a multiscale structure with hierarchical levels from nano to microscale, which comprises collagen fibrils and biological apatite (BAp).^1^ The hierarchical bone tissues exhibit anisotropic properties originating from the collagen fibril orientation and the direction of the *c*-axis of BAp crystals.^2,3^ Several researchers have focused on the distribution and orientation BAp in bone tissues, using X-ray, neutron and electron diffraction techniques.^4–9^ Wenk *et al.* reported that preferred orientation of hydroxyapatite (HAp) crystallites in two mineralized tissue fragments was investigated using synchrotron X-rays.^5^ The tissues exhibited significant crystalline alignment with *c*-axis, which preferentially aligned parallel to the long axis of the bone. Ziv *et al.* showed the *c*-axis of HAp in bovine bone were aligned parallel to the collagen fibril axes using electron diffraction methods.^4^ In our previous review articles reported variety of the preferential alignment of the BAp *c*-axis of various type bones by the micro-beam X-ray diffractometer (μXRD) system equipped with two-dimensional detectors.^10,11^ The orientation *c*-axis of BAp crystallites analyzed by μXRD offers a new index as a bone quality parameter; which can obtain relative intensity ratio of the 002 diffraction peak to the 310 peak in the X-ray profile.^3,12^ Additionally, bone quality was defined by the National Institute of Health (NIH) in 2000 as parameters contributing to bone strength independent of bone mineral density (BMD).^13^ The mechanical properties of bone tissue is strongly correlated with the degree of BAp *c*-axis orientation, which is one of the indices for bone quality.^14^ Moreover, during bone regeneration, the recovery of *c*-axis orientation of BAp, which determines bone quality, is significantly delayed compared to that of BMD, which determines bone quantity. Notably, bone quality dominates the mechanical properties of bone tissue rather than bone quantity.^12,14^ Hence, bone quality reconstruction during bone regeneration is an important factor for designing scaffolds.

Fibrous scaffolds for bone regeneration *via* electrospinning methods could be applied to a biomimetic template for damaged tissue.^15,16^ Oriented nanofiber scaffolds showed the ability of controlling cell alignment to the fiber collecting direction.^17,18^ Moreover, the cells producing collagen fibril bundles were aligned in the direction of the cell orientation. Fee *et al.* reported that, fibroblasts on the oriented nanofiber scaffolds were aligned parallel to the fibers, and their gene expression was upregulated through actin production, action polymerization, and focal adhesion formation.^19^ Additionally, oriented nanofiber scaffolds were also found to upregulate the expression of osteogenic markers, such as runt-related transcription factor (Runx-2), type I collagen, alkaline phosphatase (ALP), bone sialoprotein (BSP), and osteocalcin (OCN).^20^ Kikuchi *et al.* reported that, collagen/HAp composites showed a self-organized nanostructure similar to bone, which HAp *c*-axis of nanocrystals were parallel to the collagen fibrils.^21^ In our previous work, we reported that osteoblasts orientation induced collagen/apatite matrix alignment in bone tissue.^22,23^ Additionally, *c*-axis of BAp showed preferential alignment along the direction of osteoblast-produced collagen matrix.^22^ Our previous work highlighted that anisotropic fibrous scaffolds with microfibers exhibit controllability of osteoblasts alignment by designing their morphology.^24,25^ Thus, controlling cell alignment is an invaluable strategy for reconstructing anisotropic bone matrices, such as the *c*-axis of BAp.

This work reports a fundamental investigation on designing fibrous scaffolds for reconstructing bone quality. In order to fabricate fibrous scaffolds, poly(lactic acid) (PLLA) was chosen, since it is the most widely used biodegradable polymer in biomedical fields. In this work, PLLA fibrous scaffolds were prepared using the electrospinning method, and their morphologies were controlled by fiber collecting speed. The prepared fibrous scaffolds were evaluated for morphology, cell alignment, and bone apatite orientation.

## 2. Experimental

Fibrous scaffolds with various fiber alignments were prepared using the electrospinning method. PLLA (LACEA, Mitsui Chemical, Japan) dissolved in dichloromethane (99.5%, Nacalai Tesque) at 14 wt% was the solution used for electrospinning. In our preliminary experiments, this ratio was found to be optimal for preparing the fibrous scaffolds with micrometer-sized diameter. The prepared solution was loaded into a syringe needle (18 gauge) set at 2.5 μL s^−1^. A high-voltage supply (HARb-40P0.75, Matsusada Precision Inc., Japan) was used to apply 16 kV to the needle tip. The distance between the needle tip and drum collector was maintained at 200 mm. The drum collector (*φ* 60 mm) was rotated at 0.1–10.0 m s^−1^ (30–3000 rpm). The obtained fibrous scaffolds were denoted as PLLA_*x*, where *x* is the fiber collecting speed. The morphology of PLLA_*x* was observed by field emission gun electron microscopy (SEM, JSM-6500, JEOL, Japan) after coating with amorphous osmium layer using an osmium coater (Neoc CS, Meiwafosis, Japan). Fiber diameter, and angle (*θ*) between the fiber and collector rotation direction were measured using the ImageJ software (NIH, USA).

Primary osteoblasts were isolated form newborn mouse calvariae as described in our previous reports.^26,27^ Calvariae from newborn C57BL/6 mice were excised under aseptic conditions. The calvariae were placed in ice-cold alpha-minimum essential medium (α-MEM, Invitrogen), and then fibrous tissues around the bone were gently removed. Subsequently, the calvariae were subjected to a series of collagenase (Wako Pure Chemical, Japan)/trypsin (Nacalai Tesque, Japan) digestions at 37 °C for 15 min each. Since the fibroblasts were mixed, the supernatants of first and 2nd digests were discarded.^28^ The supernatants of 3rd–5th digests were neutralized with α-MEM and pooled. The pooled solution was filtered using a 100 μm mesh. The filtrate was centrifuged (1500 rpm, 5 min, 25 °C), and the resulting pellet was resuspended in α-MEM. All animal procedures were performed in accordance with the Guidelines for Care and Use of Laboratory Animals of Osaka University and approved by the Animal Ethics Committee of Osaka University Committee for Animal Experimentation.

PLLA_*x* with 8 mm diameter was soaked in 70% ethanol for 30 seconds and subsequently dried under UV light for 30 min for sterilization. The cells were cultured in α-MEM containing 10% fetal bovine serum (FBS, Invitrogen). PLLA_*x* was then placed into 48 well plates, and primary osteoblasts were seeded by adding 0.5 mL of medium containing cells at a concentration of 3 × 10^4^ cells per mL. The culture medium was replaced after day 1 and 3, and subsequently twice a week. After culturing for a week, the media was supplemented to achieve final concentrations of 50 μg mL^−1^ ascorbic acid (Sigma-Aldrich), 10 mM β-glycerophosphate (Sigma-Aldrich), and 50 nM dexamethasone (MP Bioscience). PLLA_*x* was analyzed for cell alignment and evaluation of calcified tissues after 3 days and 4 weeks of culture, respectively.

The primary osteoblasts were cultivated for 3 days on PLLA_*x* (*n* = 3). The cells were then fixed with 4% formaldehyde in PBS for 20 min and washed 3 times with PBS-0.05% Triton X-100 (PBST). Subsequently, the cells were incubated in PBST containing 1% normal goat serum for 30 min to block nonspecific antibody binding sites, and then incubated with mouse monoclonal antibodies against vinculin (Sigma-Aldrich) at 4 °C for 12 h. After washing 3 times with PBST, the cells were incubated with Alexa Fluor® 546-conjugated anti-mouse IgG (Invitrogen), followed by Alexa Fluor® 488-conjugated phalloidin (Invitrogen). Finally, the cells were washed 3 times, and mounted in Fluoro-KEEPER antifade reagent with DAPI (Nacalai Tesque). Fluorescent images were obtained using a fluorescence microscope (BZ-X700, Keyence, Japan). Cell orientation angle (*θ*) against the collector rotation direction was analyzed using Cell Profiler software (Broad Institute Cambridge).

PLLA_*x* (*n* = 7) cultivated for 4 weeks were fixed with 4% formaldehyde in PBS for 20 min. Bone apatite crystals produced by primary osteoblasts were analyzed by μXRD system (R-Axis BQ, Rigaku, Japan) equipped with a transmission optical system (Mo-Kα radiation, 50 kV, 90 mA) and an imaging plate (storage phosphors) (Fuji Film, Tokyo, Japan) place behind the specimen. Detailed conditions for measurement have been described in the previous paper.^29,30^ In this work, the incident beam focused into a diameter of 800 μm was used and diffraction data were collected for 1200 s. The preferred orientation of apatite *c*-axis was evaluated as the relative intensity ratio of the 002 diffraction peak to the 310 peak, which was measured in parallel to the collector rotation direction of the scaffolds. The intensity of 002 and 310 peaks in XRD profile were obtained from patterns reconstructed using multipeak fitting package (Igor Pro, WaveMetrics).

The orientation order parameter FD and CD was calculated to evaluate the degrees of fiber and cell alignment.^31^ This system was derived by using a distribution function *n*(*θ*), which is defined as the number of measured fibers or cells at the angle *θ*. The expected value of the mean square of cosine 〈cos^2^*θ*〉 and FD and CD is calculated as follows:1
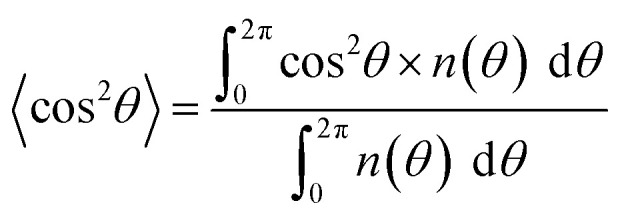

2 FD or CD = 2(〈cos^2^*θ*〉 − 0.5)

The degree of fiber or cell alignment, FD or CD takes a value ranging from −1 (fiber or cell were completely aligned perpendicular to the collector rotation direction), 0 (fiber or cell were oriented randomly), to 1 (fiber or cell were completely aligned parallel to the collector rotation direction).

Statistical comparisons between the two means were performed using a two-tailed unpaired Student's *t*-test followed by a *F*-test for homoscedasticity. *p* < 0.05 was considered significant. PLLA_0.1 was selected for comparison group, which the fibers randomly oriented.

## 3. Results and discussion

**Fig. 1:**
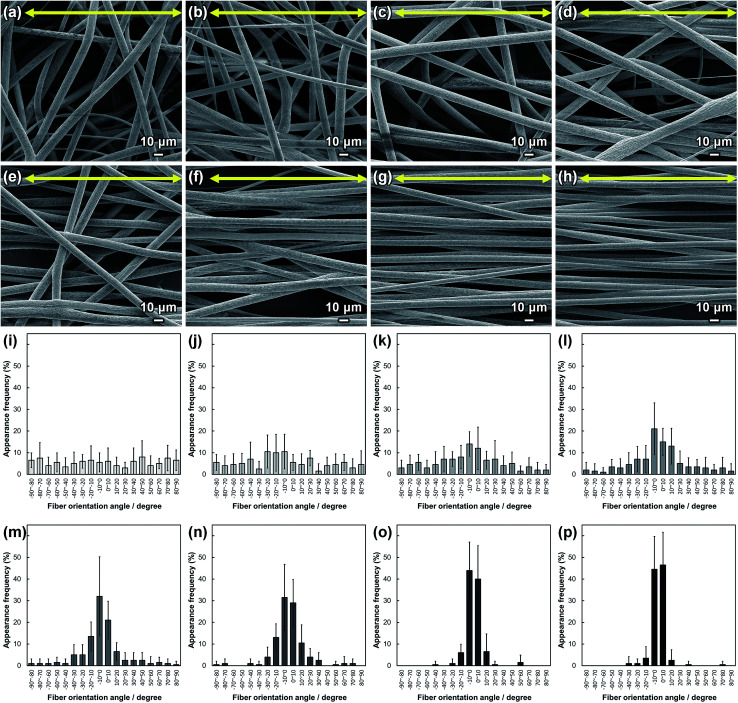
(a–h) SEM images of PLLA_*x*; *x* = (a) 0.1, (b) 1.0, (c) 2.0, (d) 3.0, (e) 4.0, (f) 5.0, (g) 7.5, and (h) 10.0. Yellow arrows indicate the collector rotation direction (0°). (i–p) Fiber orientation angle histograms for PLLA_*x*; *x* = (i) 0.1, (j) 1.0, (k) 2.0, (l) 3.0, (m) 4.0, (n) 5.0, (o) 7.5, and (p) 10.0. Error bars represent standard deviation.

**Fig. 2:**
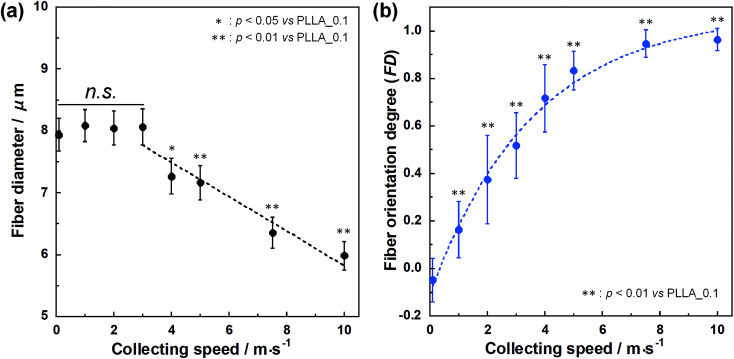
(a) Fiber diameters of PLLA_*x*. Dashed line represents the correlation between fiber diameter and fiber collecting speed (*x* = 3–10), and *n.s.* represents no significant difference. (b) Fiber orientation degree (FD) of PLLA_*x*, and dashed line represents correlation between FD and fiber collecting speed. Error bars represents standard deviation.

SEM images of PLLA_*x* are shown in Fig. 1(a–h), and their fiber orientation angle histograms are presented in Fig. 1(i–p). The fiber orientation angle was distributed at a center of 0 degree, and the breadth decreased with increasing fiber collecting speed. Fiber diameter of PLLA_*x* is shown in Fig. 2(a), and the calculated FD value of PLLA_*x* is shown in Fig. 2(b). During the electrospinning process, fibers were formed by the creation and elongation of an electric field fluid jet.^32^ The velocities of the jets were measured in the range of 0.5 to 5.0 m s^−1^*via* high framerate video camera.^32^ In this work, fiber collecting speed was set between 0.1 and 10.0 m s^−1^, and the jet was single and stable throughout the fabrication. Fiber diameter of PLLA_*x* with the collecting speed *x* = 0.1–3.0 showed no significant difference, and that with *x* = 4.0–10.0 decreased with increasing collecting speed. In case of the collecting speed *x* > 3.0, the fluid jets were stretched during the fabrication of scaffolds, since velocities of fiber collecting speed were larger than the fluid jets formed in the present electrospinning conditions. Specifically, the fiber diameters of PLLA_*x* with *x* > 3.0 showed negative linear correlation with the collecting speed (*p* < 0.01, *R*^2^ = 0.94), and the diameter exhibited significant smaller than that of PLLA_0.1. In contrast, the fiber collecting speed of *x* ≤ 3.0 is smaller than the velocity of the fluid jet in this work; thus, the fiber diameters showed no significant difference for PLLA_*x* with *x* ≤ 3.0. FD of PLLA_0.1 was −0.05 ± 0.09, which indicates that the fibers were randomly oriented. FD of PLLA_10 was 0.96 ± 0.05, which shows that almost all fibers were aligned parallel to the collector rotation direction. FD of PLLA_*x* with *x* > 0.1 showed significant larger values compare with PLLA_0.1, which the fibers randomly oriented. Moreover, FD of PLLA_*x* showed good correlation with the fiber collecting speed using negative exponential decay function (*R*^2^ = 0.99), indicating that the morphology of fibrous scaffolds, such as fiber diameter and alignment, can be controlled by the condition of electrospinning, *e.g.*, fiber collecting speed.

**Fig. 3:**
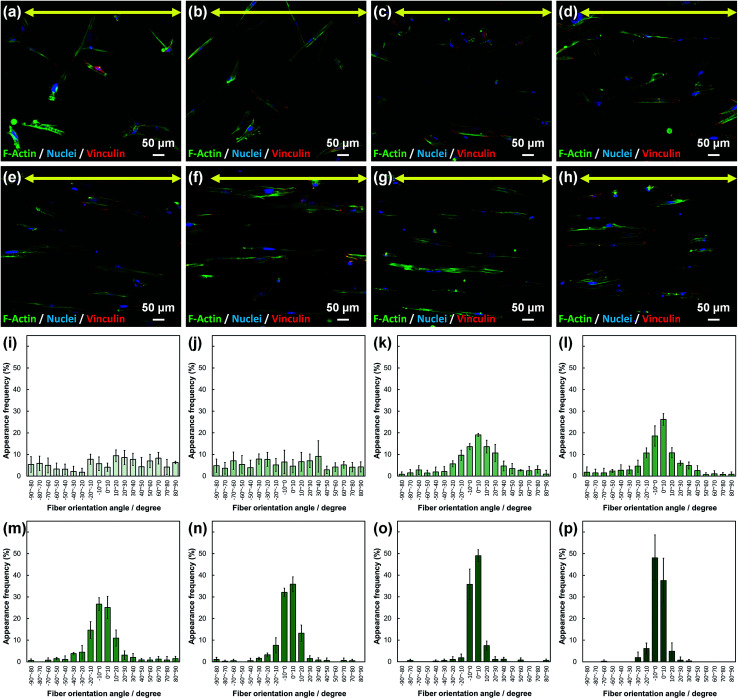
(a–h) Fluorescence images of primary osteoblasts cultured on PLLA_*x*; *x* = (a) 0.1, (b) 1.0, (c) 2.0, (d) 3.0, (e) 4.0, (f) 5.0, (g) 7.5, and (h) 10.0. Yellow arrows indicate the collector rotation direction (0°). Green: F-actin, blue: nuclei, and red; vinculin. (i–p) Cell orientation angle histograms for PLLA_*x*; *x* = (i) 0.1, (j) 1.0, (k) 2.0, (l) 3.0, (m) 4.0, (n) 5.0, (o) 7.5, and (p) 10.0. Error bars represent standard deviation.

**Fig. 4:**
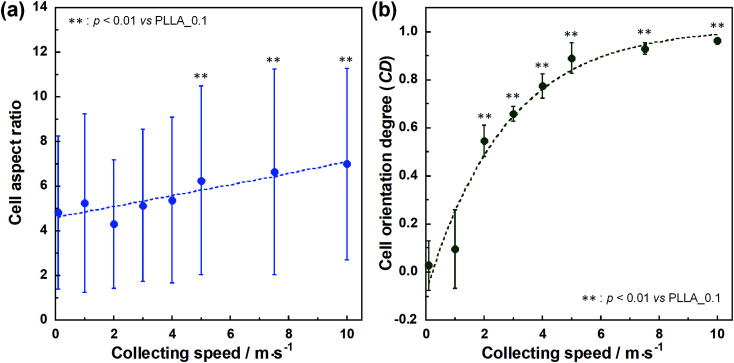
(a) Cell aspect ratio, (b) cell orientation degree (CD) on PLLA_*x*, and dashed lines represent correlation between the fiber collecting speed and cell aspect ratio and CD, respectively. Error bars represents standard deviation.

**Fig. 5:**
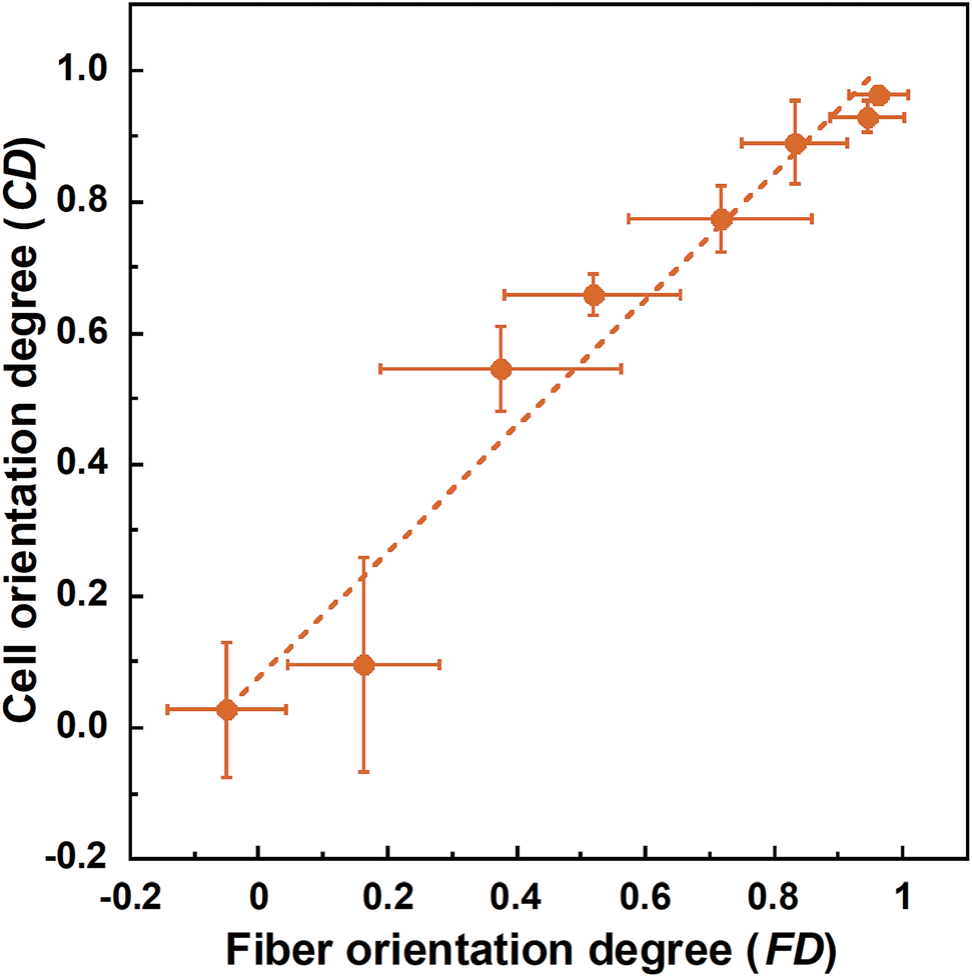
Correlation between fiber orientation degree (FD) and cell orientation degree (CD). Error bars represents standard deviation.

Cell fluorescence images on PLLA_*x* are shown in Fig. 3(a–h), and their cell orientation angle histograms are shown in Fig. 3(i–p). The breadth of distribution for cell orientation angle decreased with increasing fiber collecting speed, similar to the fiber orientation angle distribution. Sun *et al.* reported that cells on the fibrous scaffolds showed different adhering behavior depending on the diameter of fiber: a single fiber for diameters larger than 10 μm, and several fibers with spreading for diameters smaller than 10 μm.^33^ Our previous work also showed similar tendency in the fibrous scaffolds with diameters > 6 μm, indicating that cells adhered on a single fiber.^24,25^ In this work, fiber diameter of PLLA_10, which is the smallest fiber diameter in PLLA_*x*, was approximately 6 μm; the cells on PLLA_*x* can adhere to a single fiber surface. Cell aspect ratio on PLLA_*x* is shown in Fig. 4(a), and the ratio showed a linear correlation with the fiber collecting speed (*p* < 0.01, *R*^2^ = 0.82). In case of PLLA_*x* with decreasing fiber collecting speed, the fibers showed larger number of cross points and the angles between the fibers were larger, too. The cells on PLLA_0.1, where the fibers were randomly arranged, were spread and adhered on several fibers. However, those on PLLA_10 were adhered to single fiber surfaces, elongated in the longitudinal direction of the fiber. The cell aspect ratios on PLLA_*x* with *x* ≥ 5.0 exhibit significant larger values compare with PLLA_0.1, due to the cells adhered on single fiber. This is caused by the fibers in the scaffolds were elongated and aligned during the electrospinning process, and followed decrease number of cross points. Thus, the aspect ratio of cells on PLLA_*x* increased with increasing fiber collecting speed. The calculated CD on PLLA_*x* is shown in Fig. 4(b). CD of PLLA_0.1 was 0.02 ± 0.10, while that of PLLA_10 was 0.96 ± 0.01, indicating that the cells were random and parallel to the collector rotation direction, respectively. CD of PLLA_*x* with *x* > 1.0 showed significant larger values compare with PLLA_0.1, which the fibers randomly oriented. Additionally, CD and fiber collecting speed showed a good correlation by negative exponential decay function (*R*^2^ = 0.98). Moreover, CD and FD showed linear correlation (*p* < 0.01, *R*^2^ = 0.95), as shown in Fig. 5. That is, cell alignment was successfully controlled by the morphology of the fibrous scaffolds, such as fiber alignment.

**Fig. 6:**
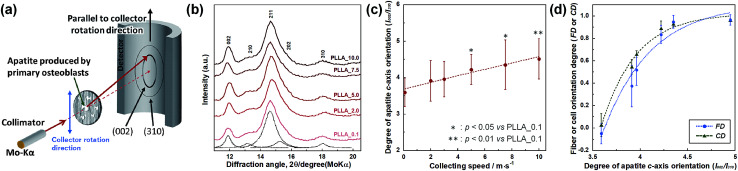
(a) Schematic illustration of the analysis of apatite orientation using transmission μXRD system. Preferential orientation of the *c*-axis of apatite crystals was analyzed with integrated intensity ratio of 002/310 in X-ray profile. (b) X-ray profiles of apatite produced by primary osteoblasts on PLLA_*x*, which profiles were obtained parallel to the collector rotation direction of the scaffolds. Dotted line represent the Lorentzian curves of each peaks, which represents reconstructed pattern of PLLA_0.1. (c) Correlation between integrated intensity ratio of *I*_002_/*I*_310_, *i.e.* degree of apatite *c*-axis orientation along the collector rotation direction, and the fiber collecting speed. (d) Correlation between degree of apatite *c*-axis orientation and FD, and CD. Error bars represents standard deviation.

Preferential orientation of the *c*-axis of apatite crystals was analyzed by μXRD system, and schematic illustration of analysis shown in Fig. 6(a). X-ray profiles of apatite produced by primary osteoblasts on PLLA_*x* cultured for 4 weeks were showed in Fig. 6(b). PLLA_*x* showed the peaks corresponding to hydroxyapatite (ICCD card: 74-0566). The obtained X-ray profiles were fitted with Lorentzian functions; the dotted lines were reconstructed peaks of PLLA_0.1, which showed representative example of PLLA_*x*. The degree of preferential orientation of the *c*-axis in the apatite crystals was determined as the relative intensity ratio of the 002 diffraction peak to the 310 peak in the X-ray profile. This was previously reported as a suitable index for evaluating apatite orientation.^3,9,12,29,30^ The degree of apatite *c*-axis orientation (*I*_002_/*I*_310_) of PLLA_*x* is shown in Fig. 6(c), with a linear correlation with the fiber collecting speed (*p* < 0.01, *R*^2^ = 0.96). *I*_002_/*I*_310_ values of PLLA_*x* with *x* > 5.0 showed significant larger values compare with PLLA_0.1, which the fibers randomly oriented. Moreover, FD and CD showed good correlation with the degree of apatite *c*-axis orientation by negative exponential decay function (*vs.* FD: *R*^2^ = 0.98, *vs.* CD: *R*^2^ = 0.99), as shown in Fig. 6(d). In our previous work, collagen matrix produced by aligning primary osteoblasts were oriented in the direction of cellular alignment, and the *c*-axis of the deposited apatite crystals indicated preferential alignment along the direction of the collagen matrix.^22^ Consequently, *c*-axis orientation of bone apatite produced by primary osteoblasts on PLLA_*x* could be controlled by the morphology of fiber alignment, *i.e.*, fiber collecting speed, which is similar to those of FD and CD. Therefore, morphology for the designed fibrous scaffolds in this work has successfully controlled cell alignment, as well as the direction of calcification, *i.e.*, bone quality.

## 4. Conclusion

Designing fibrous scaffolds for reconstruction of bone quality was investigated. FD of PLLA_*x* increased with increasing fiber collecting speed. Similarly, CD on PLLA_*x* increased with increasing fiber collecting speed. Thus, cell alignment on the fibrous scaffolds can be controlled by their morphology, such as fiber alignment. Furthermore, the apatite *c*-axis orientation degree, which is produced by primary osteoblasts, also increased with increasing fiber collecting speed. Therefore, designing fiber alignment of fibrous scaffolds with larger FD is more effective for bone quality reconstruction. These fundamental investigations are crucial to achieve further breakthrough in the research on regeneration of bone quality.

## Conflicts of interest

Authors have no conflict of interests to declare.

## Supplementary Material
